# Distinct angiogenesis roles and surface markers of early and late endothelial progenitor cells revealed by functional group analyses

**DOI:** 10.1186/1471-2164-14-182

**Published:** 2013-03-15

**Authors:** Cheng-Chung Cheng, Shing-Jyh Chang, Yu-Neng Chueh, Tse-Shun Huang, Po-Hsun Huang, Shu-Meng Cheng, Tsung-Neng Tsai, Jaw-Wen Chen, Hsei-Wei Wang

**Affiliations:** 1 Division of Cardiology, Department of Internal Medicine, Tri-Service General Hospital, National Defense Medical Center, Taipei, Taiwan; 2 Institute of Microbiology and Immunology, National Yang-Ming University, Taipei, Taiwan; 3 VGH Yang-Ming Genome Research Center, National Yang-Ming University, Taipei, Taiwan; 4 School of Medicine, National Yang-Ming University, Taipei, Taiwan; 5 Cardiovascular Research Center, National Yang-Ming University, Taipei, Taiwan; 6 Division of Cardiology, Department of Medicine, National Taipei Veterans General Hospital, Taipei, Taiwan; 7 Department of Obstetrics and Gynecology, Mackay Memorial Hospital, Hsin Chu, Taiwan; 8 Department of Education and Research, Taipei City Hospital, Taipei, Taiwan

## Abstract

**Background:**

Endothelial progenitor cells (EPCs) play a fundamental role in post-natal vascular repair. Currently EPCs are defined as either early and late EPCs based on their biological properties and their time of appearance during *in vitro* culture. EPCs are rare and therefore optimizing isolation and culture is required before they can be applied as part of clinical therapies.

**Results:**

We compared the gene profiles of early/late EPCs to their ancestors CD133+ or CD34+ stem cells and to matured endothelial cells pinpointing novel biomarkers and stemness genes. Late EPCs were enriched with proliferation and angiogenesis genes, participating in endothelial tubulogenesis and hence neovascularization. Early EPCs expressed abundant inflammatory cytokines and paracrine angiogenic factors, thereby promoting angiogenesis in a paracrine manner. Transcription factors involved in EPC stemness were pinpointed in early EPCs (MAF/MAFB) and in late EPCs (GATA6/IRF6).

**Conclusions:**

The detailed mRNA expression profiles and functional module analysis for different EPCs will help the development of novel therapeutic modalities targeting cardiovascular disease, tumor angiogenesis and various ischemia-related diseases.

## Background

Defects in angiogenesis (blood vessel growth) or in vessel repair are major complications in many diseases, such as diabetes, atherosclerosis and myocardial infarction. Post-natal neoangiogenesis relies largely on circulating endothelial progenitor cells (EPCs) [1]. The regulation of angiogenesis depends not only on the number of circulating EPC but also on their activities [1]. Current EPC definitions are based predominantly on phenotypes and biological properties. Early EPCs (eEPCs) appear early (<1 weeks) in culture dishes, whereas late EPCs appear late (2–4 weeks) and have a cobblestone-like morphology [2]. Strikingly different angiogenic properties between these two EPC subpopulations have been disclosure by angiogenesis assay: late-outgrowth EPCs but not eEPCs form vascular networks *de novo*, while late EPCs but not eEPCs are incorporated into vascular networks [3]. By way of contrast, eEPCs, but not late EPCs, indirectly augment tubulogenesis even when physically separated by a Transwell membrane, which implies that the effect is via a paracrine mechanism [3-5].

Given their involvement in pathological and physiological angiogenesis, there has been growing interest in understanding and manipulating EPCs for therapeutic purposes. Detailed molecular analysis of EPCs before and during endothelial differentiation is still lacking. Since there is no direct differentiation system available for obtaining EPCs from CD133+ stem cells (hemangioblasts), the post-natal endothelial stem cells, genomics data so far has relied on a purified subpopulation (“static”) approach. Microarray analysis has been performed on freshly isolated (day 0) human cord blood-derived endothelial progenitors (CD133 + KDR + or CD34 + KDR+), and at different time points during *in vitro* differentiation (early: day 13; late: day 27) [6]. With this information, it has been possible to identify molecular targets crucial for EPC differentiation and stemness and to test their involvement in EPC function. For example, in a follow-up study, the Notch signaling pathway was found to regulate EPC pro-angiogenic or pro-wound healing properties [7]. Recent research has applied microarray and proteomics approaches to unmask the mRNA and protein compositions of eEPC and late EPC [8]. Re-analyzing and organizing array data using an advanced systems biology approaches to better understand EPC biology will improve significantly our understanding of EPC biology.

In this study we aim to better define the roles of early and late EPCs in angiogenesis, as well as to explore some of the underlying mechanisms. AC133+/CD133+ hemangioblasts, CD34+ angioblast precursors and terminal differentiated matured ECs were included as reference cell types.

## Methods

### Isolation and cultivation of EPCs from cord blood

Fresh human cord blood was obtained from pregnant female volunteers aged 25–35, without significant disease, not receiving any medication and without any clinical diagnosis. All patients gave informed consent, and the study was approved by the local research ethics committee. The protocols of this study were consistent with ethical guidelines provided in the 1975 Helsinki Declaration. EPC isolation and characterization were done as described with minor modifications [9,10]. Cord blood mononuclear cells (MNCs) isolated by Histopaque-1077 (1.077 g/ml, Sigma, St. Louis, Missouri, USA) density-gradient centrifugation to minimize cellular blood components such as platelets. 1 × 10^7^ mononuclear cells (MNCs) were plated in 2 ml endothelial growth medium-2 (Lonza Ltd., Basel, Switzerland), with supplementation (hydrocortisone, IGF-1, human EGF, human VEGF, human FGF-B, ascorbic acid, GA-1000, heparin and 2% fetal bovine serum) in a fibronectin-coated well of a 6-well plate. After 3–5 days of 5% CO_2_, 37°C cultivation, attached eEPCs appeared and medium and nonadherent cells were then removed. Medium were changed every two days, and colonies of late EPCs (lEPCs) appeared after 2–3 weeks. The late EPCs exhibited “cobblestone” morphology and a monolayer growth pattern that is typical of mature endothelial cells at confluence. Thereafter, lEPC colonies were trypsinized and cultured on fibronectin pre-coated (5 μg/cm^2^, Millipore) wells or plates (2 × 10^4^/cm^2^) for further experiments.

### EPC characterization and tube formation assay

The antibodies used in FACS to characterize the adherent cell population were CD34 (BD Pharmingen, Franklin Lakes, NJ USA), kinase insert domain receptor (KDR)/VEGF receptor 2 (R&D system, Minneapolis, MN USA), VE-cadherin (AbD Serotec, Kidlington, UK), AC133 (CD133), platelet–endothelial cell adhesion molecule-1 (CD31; Miltenyi Biotech GmBH, Bergisch Gladbach, Germany) and CD45 (Biolegend, San Diego, CA USA). Flow cytometry was performed using a FACSCanto flow cytometer (BD Pharmingen, Franklin Lakes, NJ USA).

The *in vitro* tube formation assay was performed by thawing Matrigel at 4°C overnight, and then placed it in a 96-well plate at 37°C for 1 h to allow the matrix solution to solidify. EPCs were harvested with trypsin/EDTA, and 1 × 10^4^ EPCs were placed on Matrigel with EGM-2 medium or serum-free DMEM and incubated at 37°C for 6 h. Tubule formation was inspected under an inverted light microscope (100×). Four representative fields were taken.

For 3D angiogenesis assay, collagen type I acidic solution were mixed with 1/2 volume of basic conditioned medium with 0.2 ug/ml SDF-1α (R&D system, Minneapolis, MN USA) and solidify 30 minutes in 96-well plate at 37°C in a 5% CO_2_ incubator. 10^5^ cells per well were seeded and assayed.

### Gene expression microarray

CD133+ stem cells and CD34+ precursor, blood vessel endothelial cells (BEC), lymphatic endothelial cells (LEC) and PBMCs array data were from our previous publication [11]. GEO microarray datasets (http://www.ncbi.nlm.nih.gov/geo/) included in this study were GSE12891 [12] and GSE10856 [13] (the sources of the microarray data were summarized in Additional file 1: Table S1). Total RNA sample preparation, cRNA probe preparation, array hybridization and data analysis were done as described previously [14]. Affymetrix^TM^ HG-U133 Plus 2.0 whole genome chips were used. Batch effects were minimized by the sva (Surrogate Variable Analysis) package of the Bioconductor suite (http://www.bioconductor.org) for the R statistical programming language (http://www.r-project.org). RMA log expression units were calculated from Affymetrix GeneChip array data using the ‘affy’ package of the Bioconductor suite. The default RMA settings were used to background correct, normalize and summarize all expression values. Significant differences between the sample groups was identified using the ‘limma’ (Linear Models for Microarray Analysis) package of the Bioconductor suite, and an empirical Bayesian moderated t-statistic hypothesis test between the two specified phenotypic groups was performed [15]. To control for multiple testing errors, we then applied a false discovery rate algorithm to these *p* values in order to calculate a set of *q* values, thresholds of the expected proportion of false positives, or false rejections of the null hypothesis [16].

Heat maps were created by the dChip software (http://www.dchip.org/). Principal component analysis (PCA) was performed by the Partek Genomics Suite (http://www.partek.com/) to provide a visual impression of how the various sample groups are related. Gene annotation was performed by our ArrayFusion web tool (http://microarray.ym.edu.tw/tools/arrayfusion/) [17]. Gene Ontology database search were performed by the DAVID 6.7 Bioinformatics Resources (http://david.abcc.ncifcrf.gov/). The Euclidean distance between two groups of samples is calculated by the average linkage measure (the mean of all pair-wise distances (linkages) between members of the two groups concerned) [14]. The standard error of the average linkage distance between two groups (the standard deviation of pair-wise linkages divided by the square root of the number of linkages) is quoted when inter-group distances are compared in the text. Differential gene expression profiles were imported into the Ingenuity Pathways Analysis (IPA) software (Ingenuity Systems, Redwood City, CA, USA; http://www.ingenuity.com/) to compare the biological activities of different cell types.

## Results

### Isolation and characterization of human endothelial precursor cells

**Figure 1:**
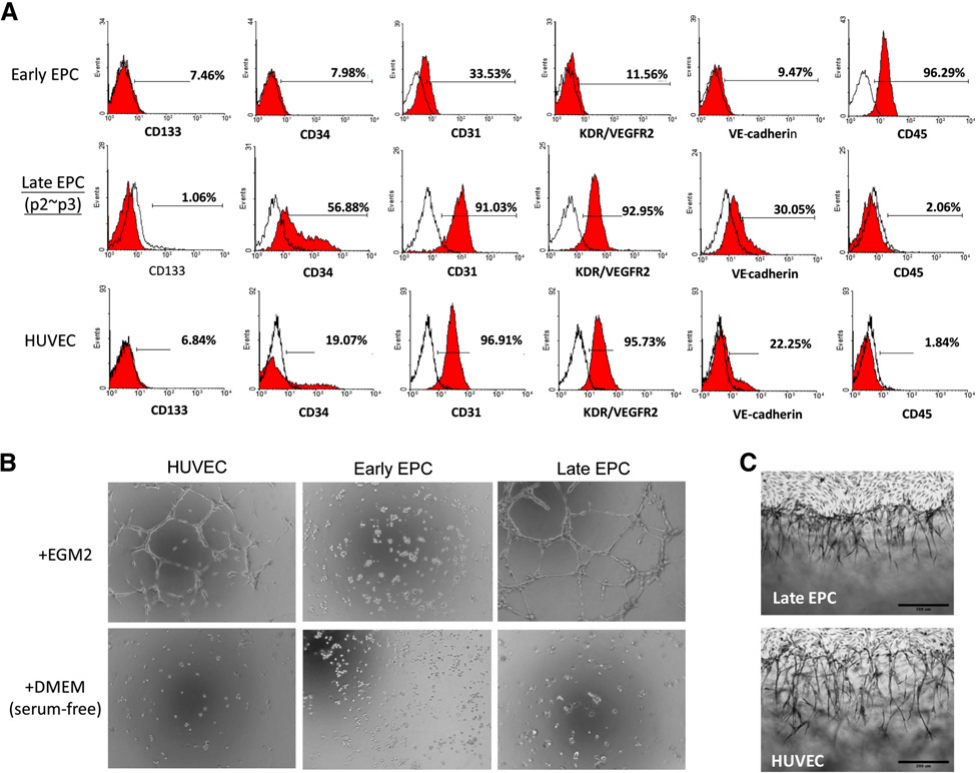
**Cultivation and characterization of early and late EPCs. **(**A**) Expression of indicated molecules in EPCs by flow cytometric analysis. (**B**) Late EPCs and matured ECs, but not early EPCs, formed tubules *in vitro*. Representative photos for *in vitro* angiogenesis are shown. (**C**) Both late EPCs and matured ECs formed vessel structures in 3D-angiogenesis assays. Bar = 200 um.

EPCs were obtained from the cord blood of healthy subjects as described [10]. The peripheral blood MNCs that were initially seeded on fibronectin-coated wells were round. After changing the medium on day 4, attached eEPCs with an elongated morphology appeared. Late EPCs with a cobblestone-like morphology similar to mature endothelial cells grew to confluence at days 14–21. The expression of cell lineage markers on the different EPCs was further validated and quantified by flow cytometry analysis. The majority of cells that were late EPCs and HUVECs expressed CD31, and KDR endothelial markers, while hematological marker CD45 was present on eEPCs (Figure 1A). In contrast, CD31 and KDR were present on only part of the isolated eEPC population (Figure 1A). An *in vitro* tube formation assay was performed using the isolated EPCs to characterize their functionality before subjecting them to genomic analysis. As expected, both HUVEC and late EPCs formed tubule networks on Matrigel, while eEPC were not able to do this (Figure 1B). Late EPCs also formed capillary-like structures in a 3D angiogenesis assay (Figure 1C).

### Microarray analysis reveals novel biomarkers for early and late EPCs

**Figure 2:**
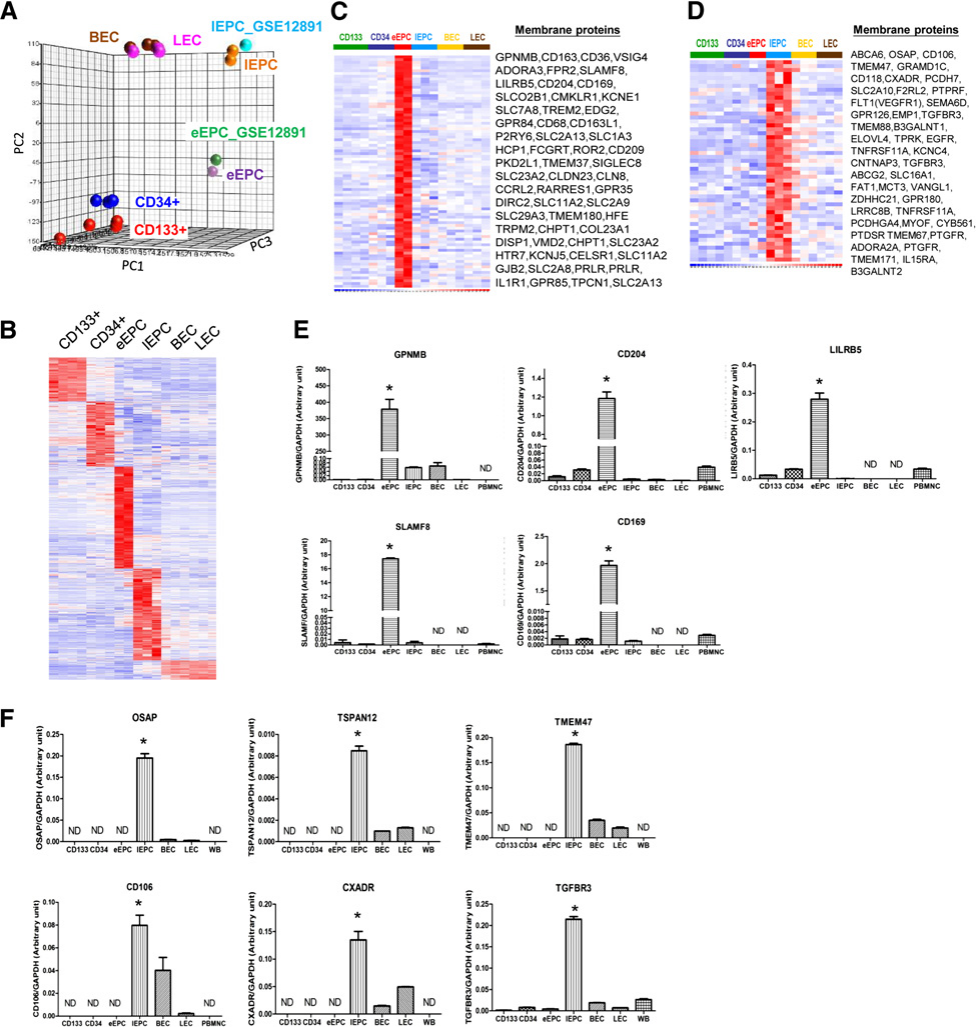
**Distinct gene expression patterns in endothelial cells of different postnatal differentiation stages. **(**A**) A principal component analysis (PCA) plot using genes differentially expressed between CD133+ stem cells and matured endothelial cells (8880 probe sets, q < 10^-4^). GSE12891: early and late EPCs from GEO data set GSE12891. Each spot represents a single array sample. (**B**) A heat map showing genes unique in each endothelial lineage cell type. Columns represent human tissue and stem cell samples, while rows represent probe sets. Genes in red: increased expression; in blue: decreased. (**C**-**D**) Cell membrane proteins specifically expressed in early (**C**) or late (**D**) EPCs according to the “Cellular Component” ontology in the GO database. (**E-F**) Mean gene expression levels of early (**E**) or late (**F**) EPC surface proteins (compared to GAPDH control; n = 3). Results are expressed as the mean ± standard deviation. ND: not detectable. *: P < 0.05 by analysis of variance (ANOVA).

Post-natal endothelial differentiation is considered to start with the AC133+/CD133+ hemangioblast stem cell population, followed by CD34+ stem/precursor cells, EPCs, and finally mature endothelial cells [11]. To access the molecular mechanisms governing the diverse behaviors of the different EPCs, as well as to help elucidate the somatic endothelial differentiation mechanisms, we analyzed EPC transcriptome profiles using whole genome chips and then compared them to those of other endothelial lineage cells obtained by our group [11]. A PCA plot using genes differentially expressed between CD133+ ancestor stem cells and matured endothelial progeny cells (n = 8880, positive false discovery rate (pFDR) q < 10^-4^) represents the differentiation hierarchical relationship (Figure 2A). PCA derived from all of the genes also showed a same conclusion (Additional file 2: Figure S1).

Some of the above 8880 genes may co-present in 2 or several cell types. To further narrow down cell type-specific genes, we filtered only genes unique (i.e., higher than in all other cell types) in each cell type. A total of 737 probe sets were found to be unique to eEPCs (q < 0.0001), 665 were unique to late EPCs (q < 0.0001), 318 were unique to CD133+ stem cells (q < 0.0001), and finally another 472 were unique to CD34+ precursors (q < 0.05). A gene expression heat map for these genes indicates the unique expression patterns present within each cell type (Figure 2B). *In silico* data was further verified by RT-qPCR. We focused first on membrane proteins in order to identify novel surface markers for early and late EPCs. CD204, CD169, GPNMB and many other membrane proteins were uniquely expressed on eEPCs (Figures 2C &2E), while genes such as CXADR, OSAP and CD106 were uniquely expressed on late EPCs (Figures 2D &2F).

### Coordinated changes in the functional groups between different EPCs

**Figure 3:**
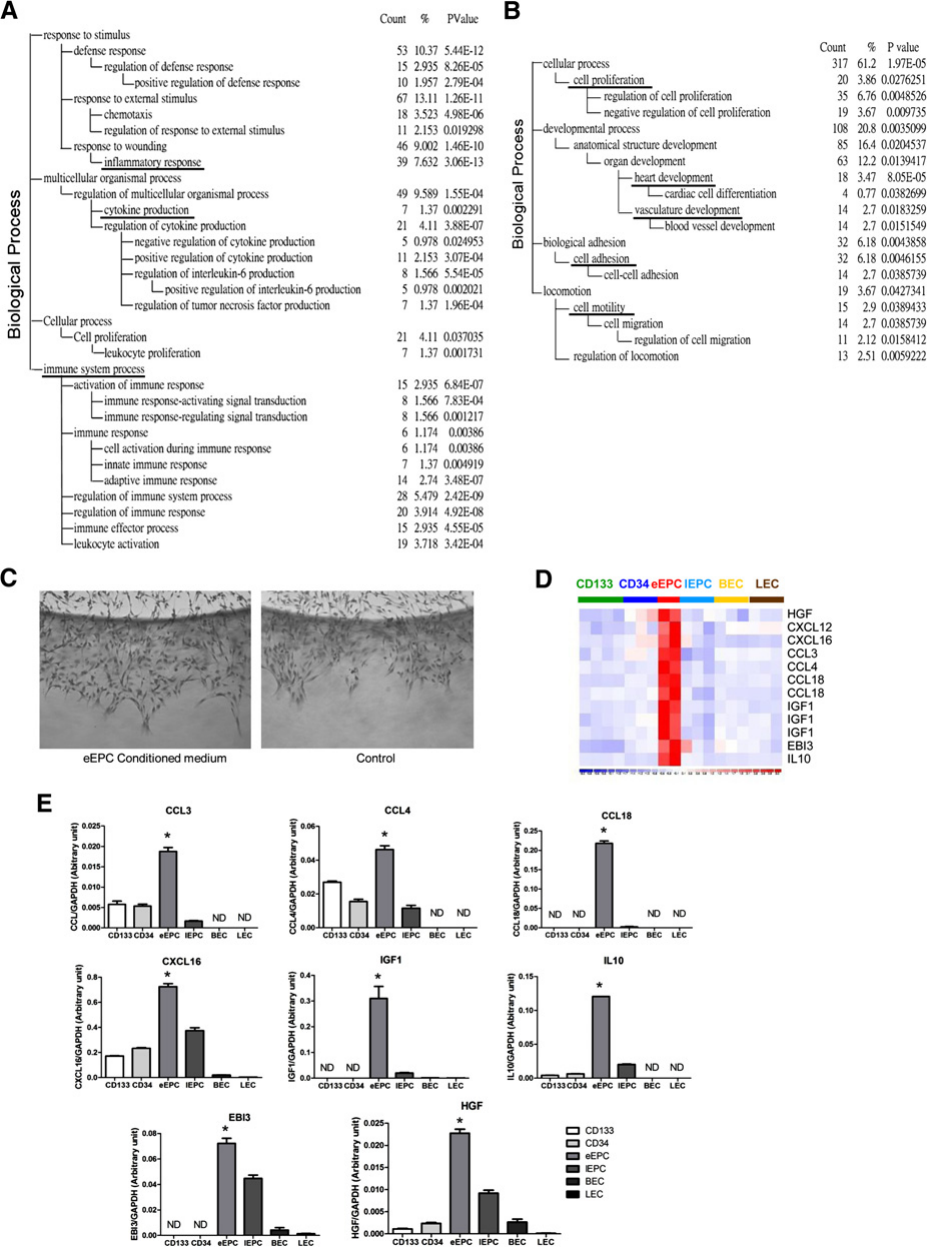
**Unique biological functions of different EPCs.** The 737 and 665 early (**A**) and late (**B**) EPC genes were subjected to a Gene Ontology (GO) database search. These categories were selected from the “Biological Process” organizing principle in the GO database (http://www.geneontology.org/). The number of genes, gene symbols, and *p* values for each category that are significantly enriched are listed (*p* < 0.05). Underlined: discussed in the text. (**C**) Culture supernatant of early EPCs increases angiogenesis ability of late EPCs in 3D-angiogenesis assays. (**D**) Paracine factors uniquely expressed in early EPCs. (**E**) Validation of array data by real-time RT-PCR (n = 3). *: P < 0.05 by analysis of variance (ANOVA).

The above gene list gave us a primary insight into the unique composition of differential EPCs but reveals little on EPC functions. To understand more how the gene expression profiles might correlate with EPC biology and to provide quantitative evidence, signature probe sets were subjected to a Gene Ontology (GO) database search to find statistically over-represented functional groups within these genes. Given that the whole human transcriptome was represented by the microarray analysis, this analysis was not biased toward the coverage of the microarray. The GO categories of the biological processes being statistically overrepresented (*p* < 0.05) among the 737 eEPC-enriched probe sets are presented in Figure 3A. The most significant biological process for eEPCs is the inflammatory response (39 genes, *p* = 3.06 × 10^-13^; Figure 3A, underlined). Cytokine production is also significantly higher in eEPCs (7 genes, *p* = 0.00229 Figure 3A). Other related predominant processes include those pertaining to chemotaxis (18 genes, *p* = 4.98 × 10^-6^), regulation of responses to external stimuli (11 genes, *p* = 0.0192) and positive regulation of defense responses (10 genes, *p* = 2.79 × 10^-4^) (Figure 3A).

In contrast, in late EPCs the most important biological processes are heart and vascular development (18 and 14 genes respectively, *p* = 8.05*10e^-4^ and 0.018; Figure 3B). BMP2, JMJD6, TGFB3, GLMN, EPAS1, and VEGFR1 all fall within these two areas. Genes involved in cell proliferation, cell adhesion and motility are also enriched in late EPCs (Figure 3B), corresponding to the motile and amplifying nature of late EPCs. Late EPCs are also enriched with Wnt signaling pathway genes according to the KEGG database (*p* = 0.018; Additional file 3: Figure S2).

The basic functional differences between endothelial lineage cells were further elucidated by comparing their gene profiles side-by-side using another function exploration tool, Ingenuity Pathway Analysis (IPA). Consistent with the GO analyses above, all cell types except eEPCs expressed significant numbers of genes involved in cardiovascular system development and function (Additional file 4: Figure S3A). Early EPCs express many more genes involved in the humoral immune response, hematological system development and function, and the inflammatory response (Additional file 4: Figure S3B-C). Canonical pathways related to innate immunity, including cytokine (such as GM-CSF and IL10) signaling, TREM1 or the toll-like receptor signaling pathway, are also specifically active in eEPCs (Additional file 4: Figure S3B-C). Early EPCs also expressed genes involved in immunity-related diseases such as systemic lupus erythematosus (SLE) and rheumatoid arthritis (RA) (Additional file 4: Figure S3C). These findings indicate that late EPCs are an attractive cell candidate for amplification *in vitro* and *in vivo* in order to induce therapeutic angiogenesis, while eEPCs should be used with caution because of their possible relationships with autoimmunity disease and allograft rejection.

### Indirect contribution of eEPCs to angiogenesis and the involvement of pro-angiogenic factors

The cytokine production and inflammatory nature of eEPCs raised a possibility that these cells contribute to angiogenesis indirectly by secreting angiogenic factors and inflammatory cytokines that aid the destruction of adjacent tissue, thereby allowing new vessels to form and to extend. It has been suggested that eEPCs assist HUVEC angiogenesis *via* an indirect paracrine manner [3-5]. We tested this hypothesis using a 3D-angiogenesis assay in which the chemotaxis potential of eEPC conditional medium (CM) was evaluated. As shown in Figure 3C, conditional medium (CM) from eEPCs induced better late EPC invasion than did the control medium. We can summarize the secretome pattern of eEPCs and this shows that eEPCs express a range of secreted factors including HGF, IL10, IGF1, CCL3, CCL4, CCL18, CXCL12, CXCL16 and IGF1 (Figure 3D). The abundant expression of these factors was verified by qPCR (Figure 3E). It has been reported that eEPCs also express various angiogenic factors, including VEGF, IL2, IL8, G-CSF and GM-CSF [18,19]. These factors were not included in our list because their expression is also abundant in other endothelial lineage cells (not shown).

### Unique transcription factors in EPCs

**Figure 4:**
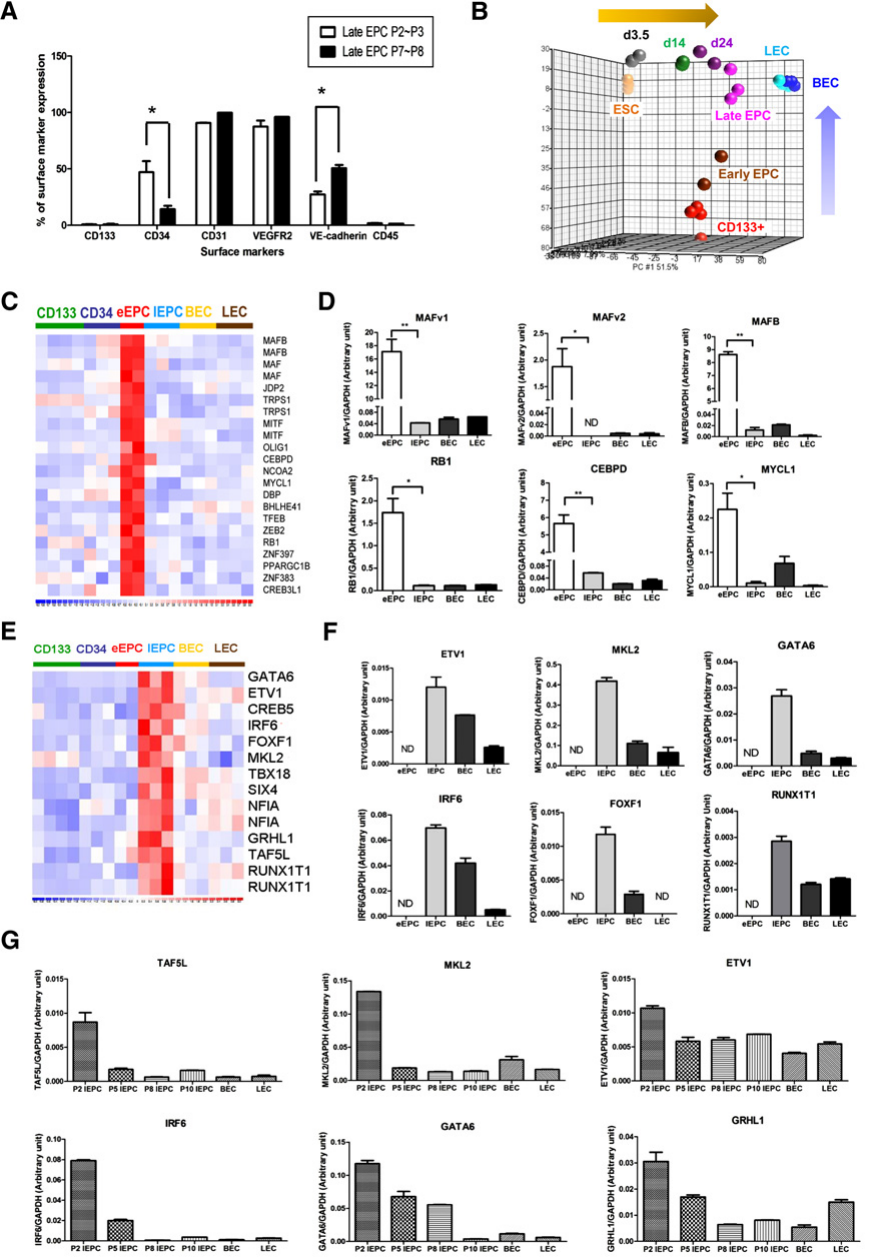
**Transcription factors enriched in the various EPCs. **(**A**) FACS data of late EPC surface antigens at different passages. (**B**) A principal component analysis (PCA) plot showing distinct differentiation pathways between embryonic and somatic endothelial differentiation (using genes differentially expressed between ESC and matured endothelial cells; 6446 probe sets, q < 10^-5^). ESC and differentiated precursors from the GEO data sets GSE19735 and GSE21668. Each spot represents a single array sample. (**C & E**) Nuclear proteins of early (C) and late (E) EPCs (defined by the “Cellular Component” ontology in the GO database) with transcription factor activities (defined by the GO “Molecular Function” ontology). (**D & F**) Validation of array data by RT-qPCR. (**G**) Expression patterns of late EPC transcription factors during *in vitro* cultivation.

One of the main problems faced when applying EPCs clinically is that there is not yet an optimal culturing system that amplifies these cells *in vitro* for a long time while still maintains their precursor status. In general, late EPCs differentiate during culturing and become senescent at around passage 15 (P15). From early passages (P2-3) to late passages (P7-8), we have observed a significant drop in CD34(+) cell numbers in late EPC population, while the percentage of VE-cadherin(+) cells is increased (Figure 4A & Additional file 5: Figure S4). To understand more about how EPC identity and stemness is maintained, we next explored the key transcription factors in each cell type. Of note, the key stemness transcription factors we filtered out may be different from those based on embryonic stem cell studies since the transcriptome changes during embryonic endothelial differentiation (orange arrow, Figure 4B) are quite different from that those of post-natal CD133+ stem cell differentiation (blue arrow, Figure 4B; PCA were performed using genes differentially expressed between ESC and matured endothelial cell). PCA derived from all of the genes also showed the same conclusion (Additional file 6: Figure S5). Under such circumstances, post-natal angiogenesis and embryonic angiogenesis should therefore, at least in part, be controlled by different regulation systems.

In eEPCs, a total of 18 nuclear transcription factors were found from array data (Figure 4C). The unique expression of two MAF family members, MAF and MAFB, was confirmed by qPCR (Figure 4D). Also unique to eEPCs are RB1, CEBPD and MYCL1 (Figure 4D). On the other hand, twelve transcription factors, including MKL2, IRF6, GATA6 and RUNX1T1, are more abundant in late EPCs (Figures 4E-F). In addition, the levels of GATA6, GRHL1 and ETV1, TAF5L, IRF6 and MKL2 dropped dramatically during the passage of late EPCs (Figure 4G), implying crucial roles in maintenance of late EPC identity.

## Discussion

EPCs play an important role in post-natal vascular repair and the maintenance of vascular homeostasis through re-endothelialization and neovascularization. The number of circulating EPCs in patient peripheral blood (PB) inversely correlates with disease prognosis, such as diabetes and cardiovascular disease (CAD) [1,20,21]. CD34, KDR, and CD133+ are considered critical markers for the outgrowth of endothelial cell–producing late EPCs, which are different from hematopoietic progenitors or leukocytes. Combinations of markers, including CD133 + CD34 + KDR+, CD34 + KDR+, or CD14 + CD34^low^, are widely used to define or select cells that express properties attributed to EPCs [22]. This approach, however, does not consider all the characteristics of EPCs and cannot distinguish eEPCs from late ones. Although CD34 + VEGFR-2 + AC133+ cells are widely accepted to represent ‘true EPCs’ in humans [23], they have never been proved to differentiate into ECs *in vivo*[22,23]. Moreover, recent studies show that mobilized adult peripheral blood CD34 + VEGFR-2 + AC133+ cells in fact represent an enriched population of CD45+ haematopoietic precursors, which do not differentiate to ECs *in vitro*[24]. The lack of known surface biomarkers for the different EPCs and the absence of a standardized protocol with regards to reagents and gating strategies may account for the widespread inter-laboratory variations in the quantification of EPC.

Another approach to quantification is to use defined culturing assays to culture both early and late EPCs and then to count colony numbers. In a model of hind limb ischaemia, late EPCs enhanced revascularization in synergy with early EPCs [4]. This strategy is also handicapped by difficulties in standardization and the prolonged assay time. In this study we have identified by mRNA profiling various novel surface markers for early and late EPCs (Figure 2). It is now possible to use new biomarkers disclosed here, together with AC133/CD34/KDR and culture assays, to direct isolate and count early and late EPCs from cord blood and peripheral blood (PB). The later point is of clinical importance since EPC number in peripheral blood has been found to correlate with disease prognosis.

It has been hypothesized that eEPC principally promote angiogenesis in a paracrine manner, while late EPC directly participate in endothelial tubulogenesis and may therefore provide the building blocks for neovascularization [3,5,18]. Paracrine factors secreted by eEPC can further prevent oxidative stress-induced apoptosis of mature endothelial cells [4,19]. HGF, VEGF, IL2, IL8, G-CSF and GM-CSF are known angiogenic factors that are secreted by eEPCs [18,19]. In addition to HGF, we discovered that eEPCs also secret abundant pro-angiogenic factors including CCL3/4/18, CXCL12/16, EBI3, IGF1 and IL10 (Figure 3). CCL3, CCL18, CXCL12/SDF-1, CXCL16, IGF1 and IL10 have been linked to pro-angiogenesis and/or coronary artery diseases [25-27]. These findings allow the development of novel angiogenic therapies that rely on the secreted growth factors delivered to sites of ischemia. Combined therapeutic angiogenesis, including the provision of local angiogenic factors and cultured eEPCs is another approach to be considered.

There are challenges to harnessing EPCs for cell therapy. One of these is their rarity (0.01-0.02 per 10^6^ mononuclear cells), which makes EPC isolation challenging. Optimization of the cultivation and amplification of EPCs is therefore required before these cells may be appropriately investigated for use in clinical therapies. It has been shown that eEPCs do not proliferate significantly *in vitro*[18]. Although late EPCs express abundant proliferation genes (Figure 3B) and can be amplified *in vitro*, the delayed outgrowth of late EPCs from culture limits their application in cases of acute ischemia (such as stroke) where there may be a limited time window for clinical benefit. Furthermore, maturation and additional differentiation occurs during propagation (Figure 4A). Late EPCs can be cultured to up to 15 passages in most cases (not shown), and the expression levels of key transcription factors (such as GATA6 and IRF6; Figure 4G) begin to alter in late EPCs during *in vitro* propagation. The unique expression pattern of the various transcription factors identified in this study suggests that they have important roles in EPC stemness and EC maturation. Controlling the levels of precursor transcription factors by gene transduction or by developing new late EPC culture cocktails should eventually benefit the clinical applications of EPCs.

## Conclusions

Our results combine mRNA profiling and gene set analysis in order to decipher the RNA expression situation at the various different stages of EPC. With this information, it will be possible to discover numerous molecular targets that are crucial for EPC differentiation and functioning. Although new research directions and hypotheses are provided by this work, careful functional studies of the genes in the context of *in vitro* and *in vivo* models of angiogenesis are still necessary to further support the clinical relevance of these exciting findings. We envision that our report will serve as a resource for future studies that aim to improve understanding of the various regulatory ultimately modulating EPC and EC activities.

## Competing interest

The authors declare no competing financial interests.

## Authors’ contributions

CCC, SJC, YNC and TSH performed data analysis and experimental validation. CCC, SJC, SMC, and TNT provided clinical materials and carried out manuscript revision. HWW, JWC and PHH conceived of the study, and contributed ideas and suggestions. CCC and HWW participated in study design and draft the manuscript. All authors read and approved the final manuscript.

## Supplementary Material

Additional file 1: Table S1 The sources of the microarray data. Click here for file

Additional file 2: Figure S1 PCA derived from all of the probesets. Click here for file

Additional file 3: Figure S2 Distribution of late EPC genes in Wnt signaling pathway. Late genes are labeled with red stars. Click here for file

Additional file 4: Figure S3 Comparative functional analysis as a basis for interpreting EPC biology. Molecular fingerprints of each cell type were subjected into IPA web tool analysis. All types except early EPC (eEPC) are enriched with genes involved in cardiovascularsystem function (**A**), while late EPC (IEPC) and matured EC are not enriched in immune response genes. (**B-C**) Unique biological modules in eEPC. Click here for file

Additional file 5: Figure S4 The changes in surface markers during cultivation of late EPC *p <* 0.05. Click here for file

Additional file 6: Figure S5 PCA derived from all of the probesets. Click here for file
