# Supplementary figures and images for: Distinct angiogenesis roles and surface markers of early and late endothelial progenitor cells revealed by functional group analyses
Source: BMC Genomics. 2013 Mar 15;14:182. doi: 10.1186/1471-2164-14-182 (PMC3652793; doi:10.1186/1471-2164-14-182)

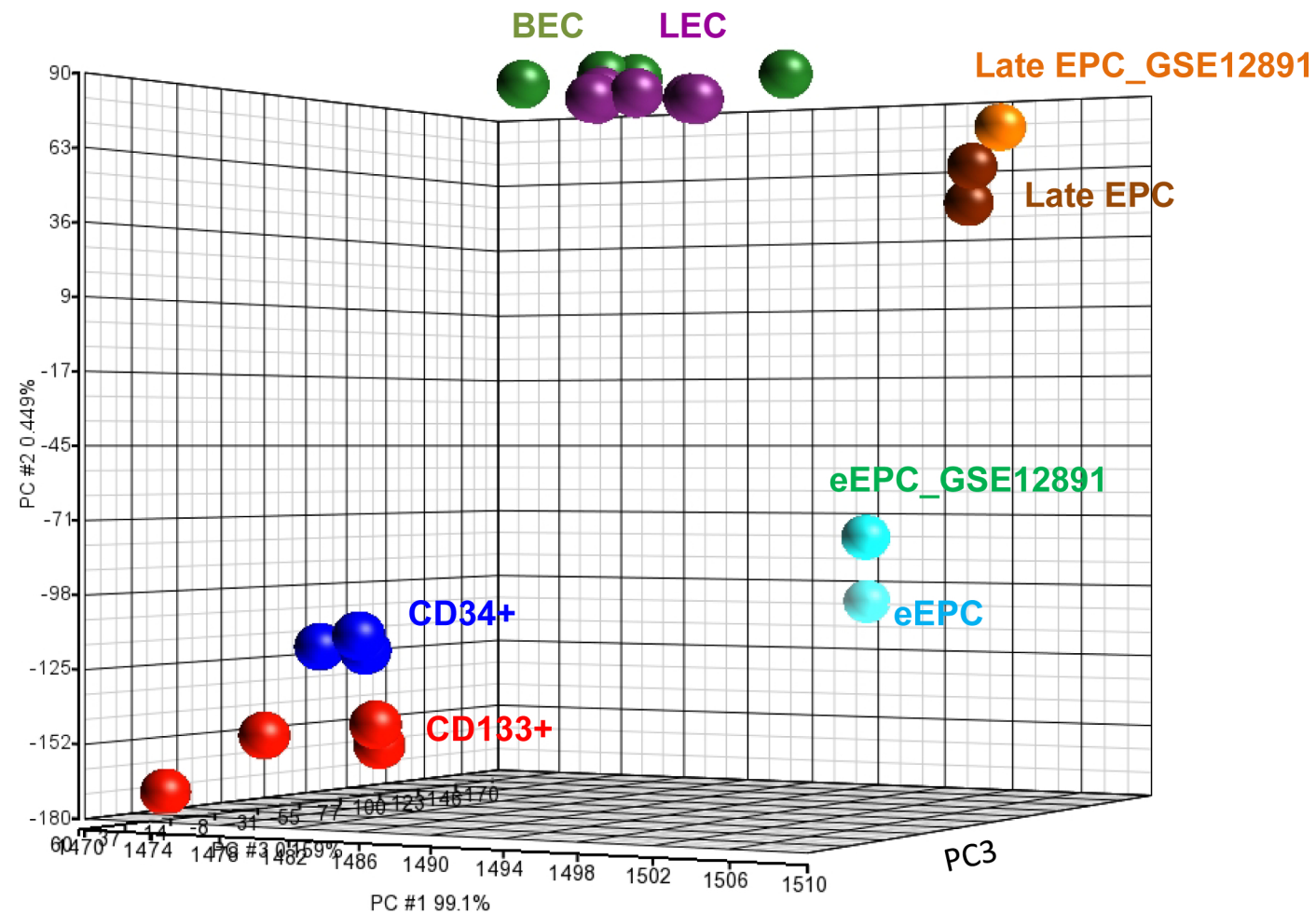

**Suppl. Figure 1.** PCA derived from all of the probesets.

Supplement: Additional file 2: Figure S1 — PCA derived from all of the probesets. [file 1471-2164-14-182-S2.pdf]

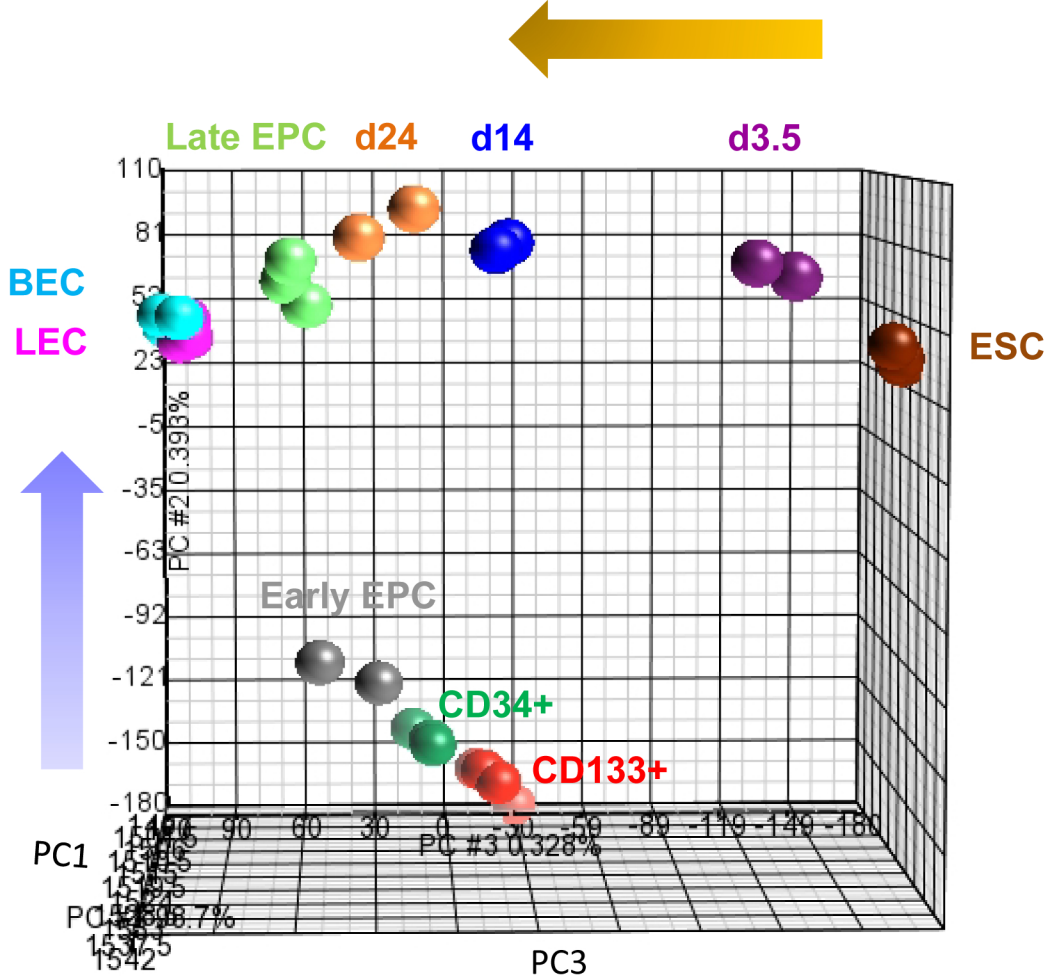

**Suppl. Figure 5.** PCA derived from all of the probesets.

Supplement: Additional file 6: Figure S5 — PCA derived from all of the probesets. [file 1471-2164-14-182-S6.pdf]
