# Supplementary material for: Distinct angiogenesis roles and surface markers of early and late endothelial progenitor cells revealed by functional group analyses
Source: BMC Genomics. 2013 Mar 15;14:182. doi: 10.1186/1471-2164-14-182 (PMC3652793; doi:10.1186/1471-2164-14-182)
Supplement: Additional file 3: Figure S2 — Distribution of late EPC genes in Wnt signaling pathway. Late genes are labeled with red stars. [file 1471-2164-14-182-S3.pdf]

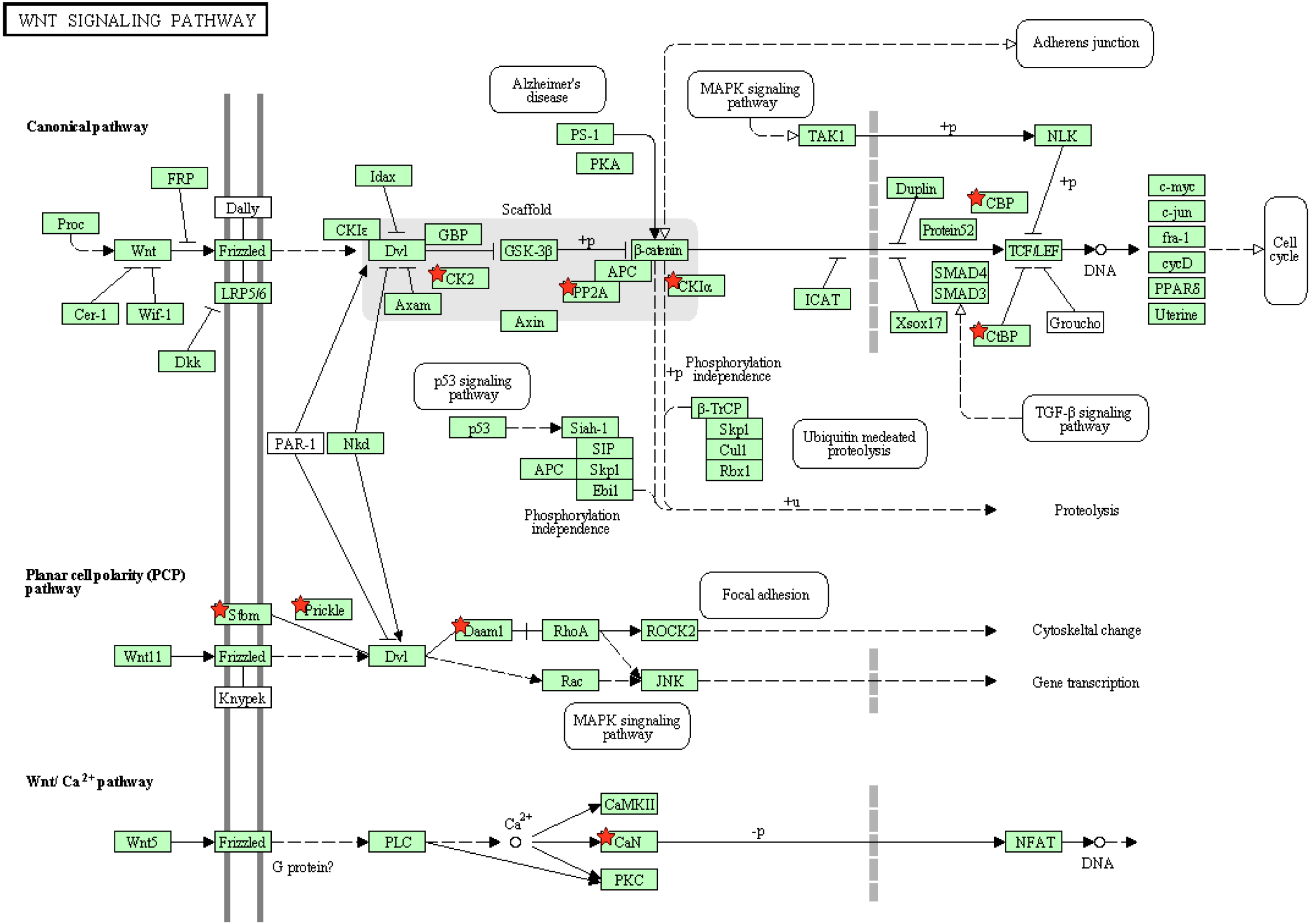

Suppl. Figure 2. Distribution of late EPC genes in Wnt signaling pathway. Late genes are labeled with red stars.
