# Supplementary material for: Distinct angiogenesis roles and surface markers of early and late endothelial progenitor cells revealed by functional group analyses
Source: BMC Genomics. 2013 Mar 15;14:182. doi: 10.1186/1471-2164-14-182 (PMC3652793; doi:10.1186/1471-2164-14-182)
Supplement: Additional file 4: Figure S3 — Comparative functional analysis as a basis for interpreting EPC biology. Molecular fingerprints of each cell type were subjected into IPA web tool analysis. All types except early EPC (eEPC) are enriched with genes involved in cardiovascularsystem function (A), while late EPC (IEPC) and matured EC are not enriched in immune response genes. (B-C) Unique biological modules in eEPC. [file 1471-2164-14-182-S4.pdf]

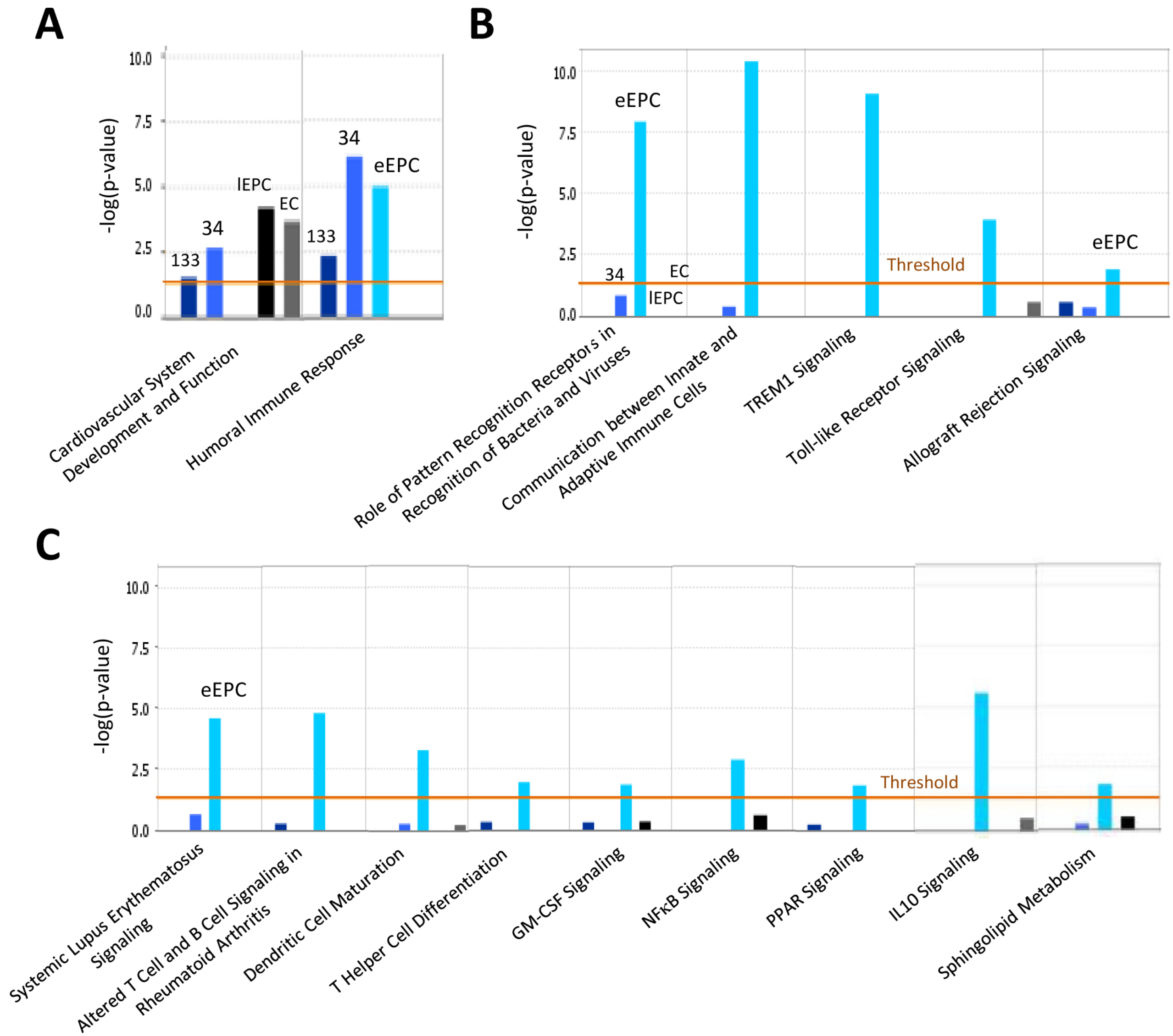

Suppl. Figure 3. Comparative functional analysis as a basis for interpreting EPC biology. Molecular fingerprints of each cell type were subjected into IPA web tool analysis. All cell types except early EPC (eEPC) are enriched with genes involved in cardiovascularsystem function (**A**), while late EPC (lEPC) and matured EC are not enriched in immune response genes. (**B-C**) Unique biological modules in eEPC.
