# Supplementary material for: Distinct angiogenesis roles and surface markers of early and late endothelial progenitor cells revealed by functional group analyses
Source: BMC Genomics. 2013 Mar 15;14:182. doi: 10.1186/1471-2164-14-182 (PMC3652793; doi:10.1186/1471-2164-14-182)
Supplement: Additional file 5: Figure S4 — The changes in surface markers during cultivation of late EPC p < 0.05. [file 1471-2164-14-182-S5.pdf]

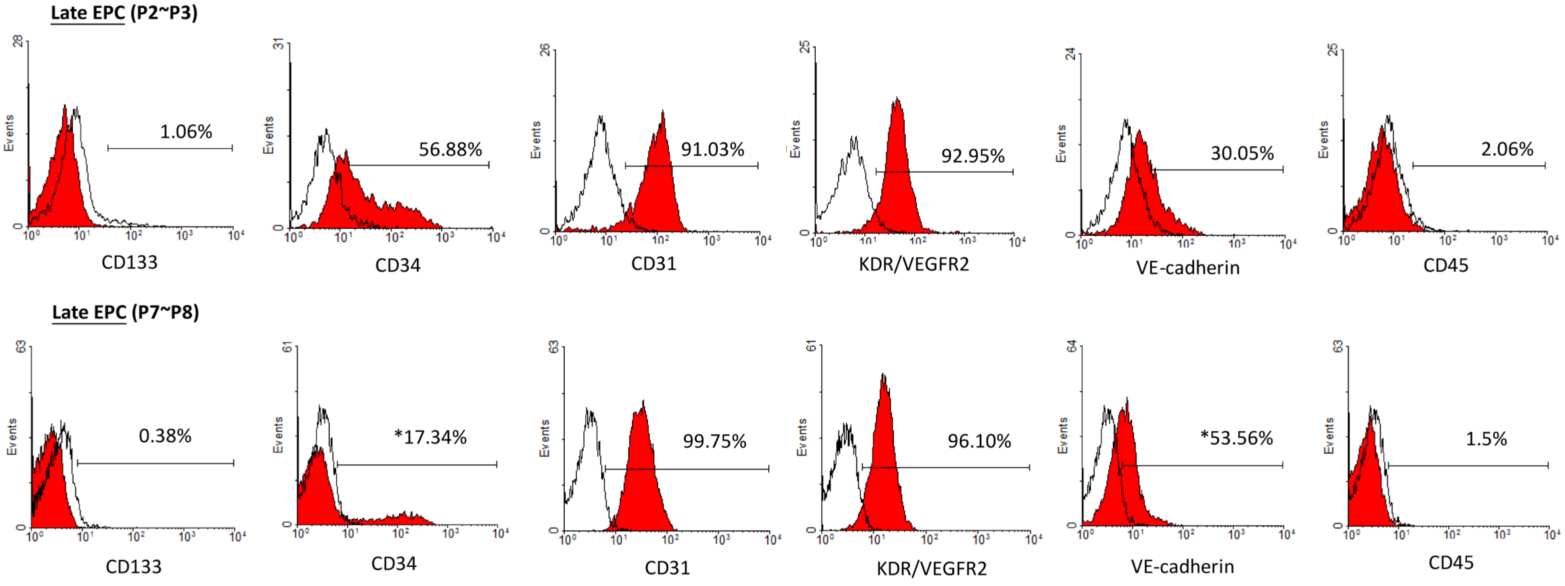

Suppl. Figure 4. The changes in surface markers during cultivation of late EPC *in vitro*. *: p<0.05.
